# Supplementary material for: Structural definition of a neutralization epitope on the N-terminal domain of MERS-CoV spike glycoprotein
Source: Nat Commun. 2019 Jul 11;10:3068. doi: 10.1038/s41467-019-10897-4 (PMC6624210; doi:10.1038/s41467-019-10897-4)
Supplement: Supplementary file 1 — Supplementary Information [file 41467_2019_10897_MOESM1_ESM.pdf]

Supplementary Information for

Structural Definition of a Neutralization Epitope on the N-terminal Domain of MERS-CoV Spike Glycoprotein

Zhou et al.

SUPPLEMENTARY FIGURES

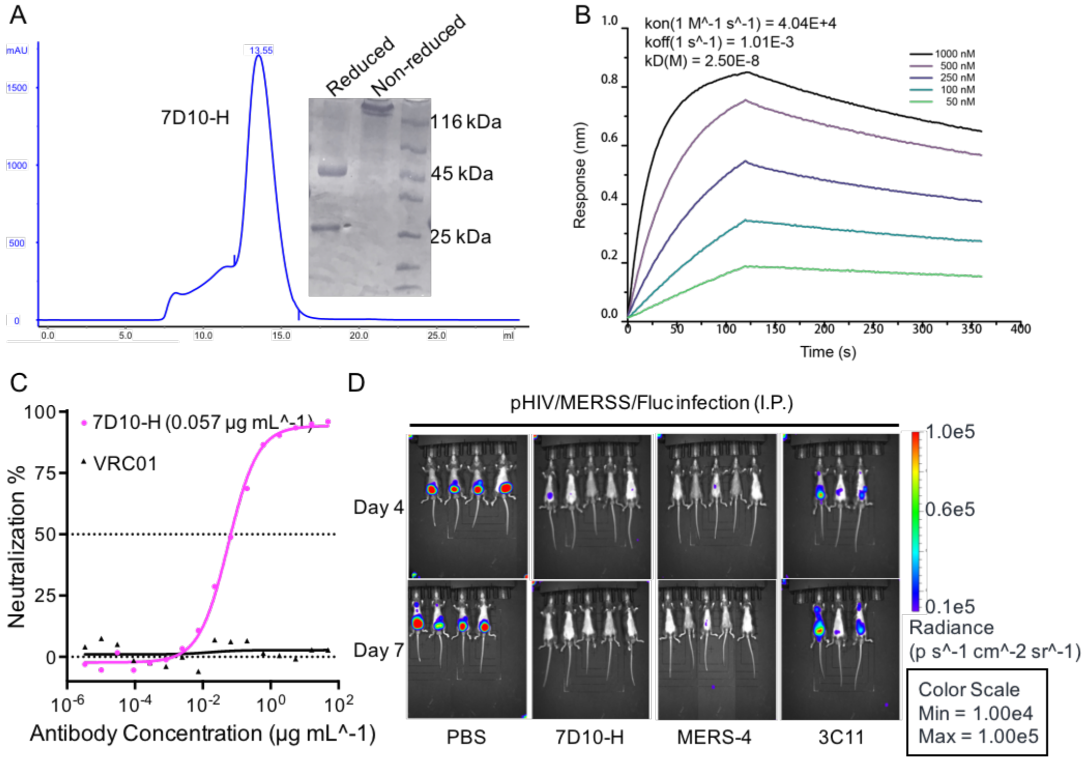

Supplementary Fig. 1 7D10-H sustaining biochemical characters and neutralizing activity. (A) Size-exclusion chromatography profiles and SDS-page analysis of 7D10-H. (B) BLI analysis of the NTD binding to 7D10-H. (C) Neutralizing test of 7D10-H against pseudotyped MERS-CoV. (D) Inhibition of pseudotyped MERS-CoV infection in R26-hDPP4 mice by the mAb 7D10-H. For evaluation of 7D10-H or MERS4, mice (N=5) were administered 200 μg per mouse of mAb I.P. For the unrelated control mAb 3C11, mice (N=3) were administered 400 μg per mouse of 3C11 I.P. and mice (N=4) were administered PBS as the negative control. And 6 h later with mAbs or PBS, all mice were challenged with pseudovirus I.P. at a dose of $1.27\times10^{7.5}$ $TCID_{50}$. On day 4 and 7 p.i., Bioluminescence imaging of the whole body was conducted and typical images are shown. Source data are provided as a Source Data file.

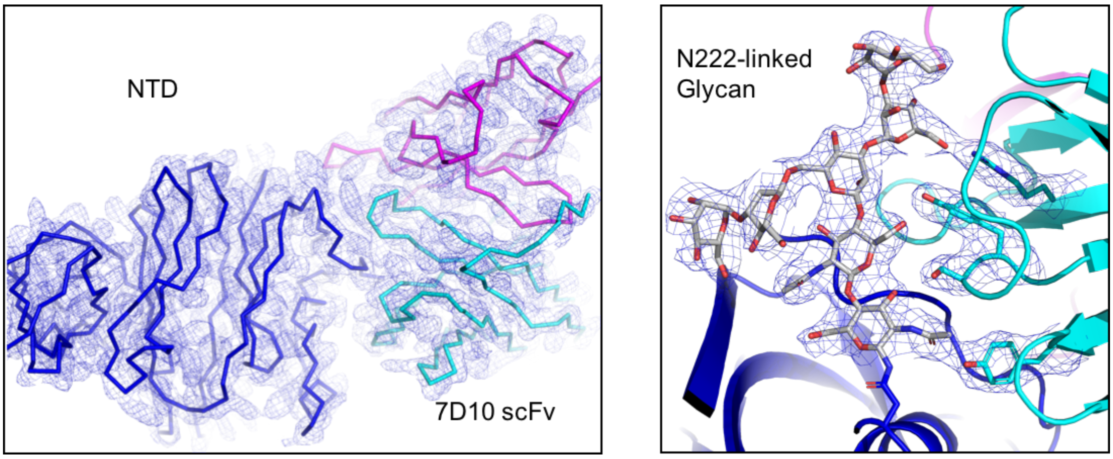

Supplementary Fig. 2 The electron density map of the NTD/7D10-scFv complex. 2Fo-Fc electron density contoured at 1.0 σ of the NTD/7D10-scFv complex (left) and the interaction between the N222-linked Glycan and 7D10-scFv (right). The NTD, N222-linked glycan on the NTD, 7D10 V_L, and 7D10 V_H are colored in blue, gray, magenta, and cyan, respectively.

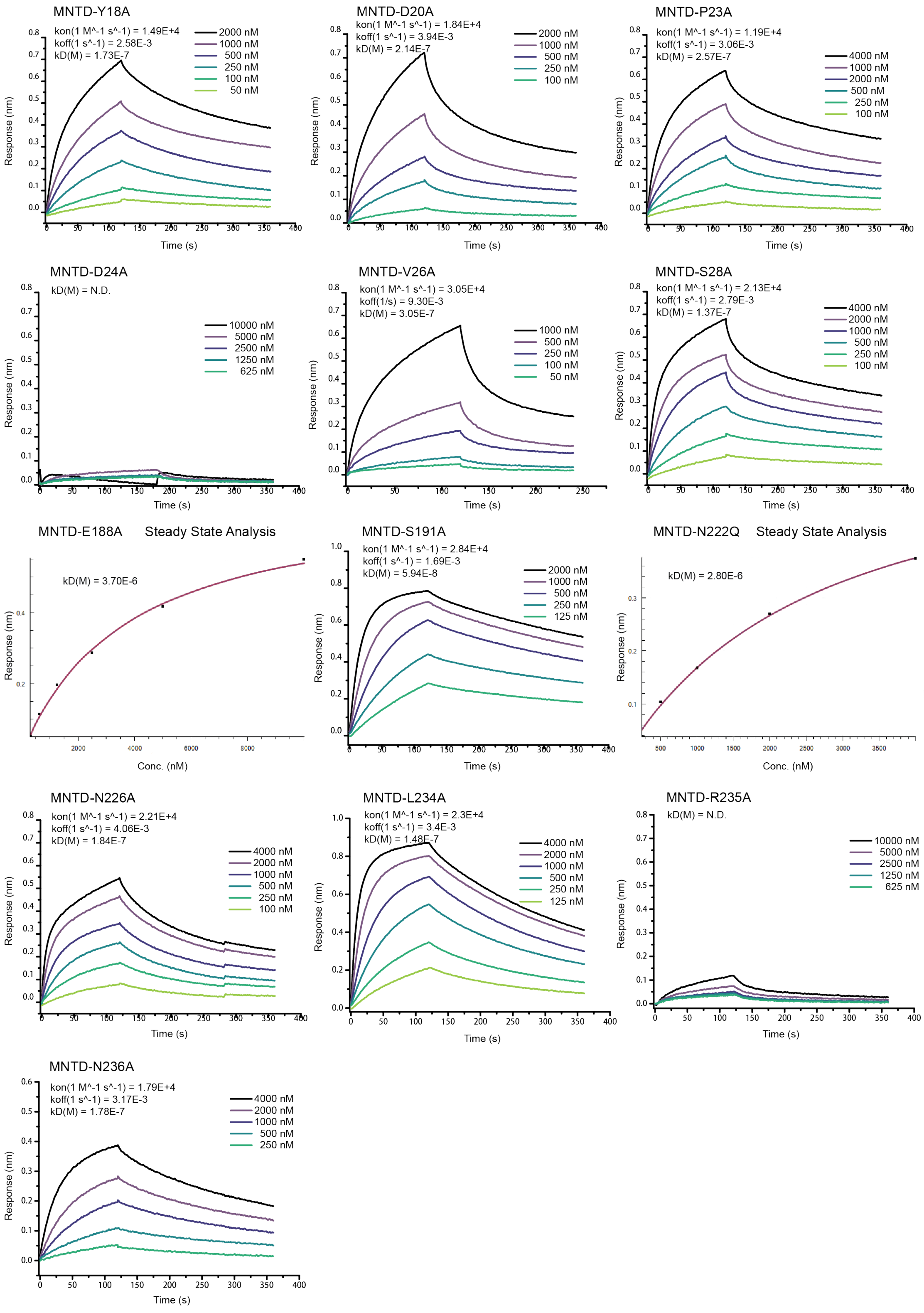

Supplementary Fig. 3 Kinetics of binding determined by BLI of 7D10-H with mutant NTD. Source data are provided as a Source Data file.

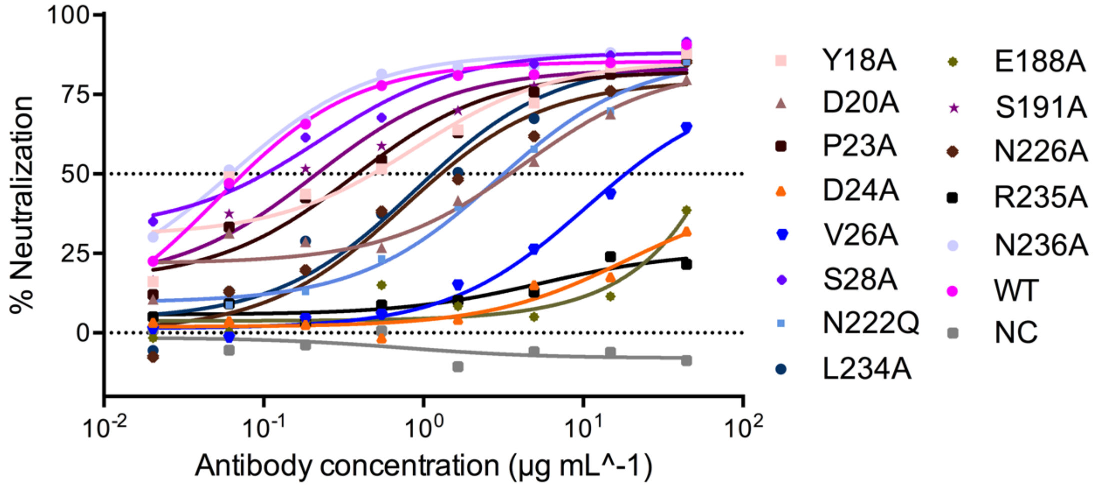

Supplementary Fig. 4 7D10-H neutralization of wild-type and mutant MERS-CoV pseudoviruses. Source data are provided as a Source Data file.

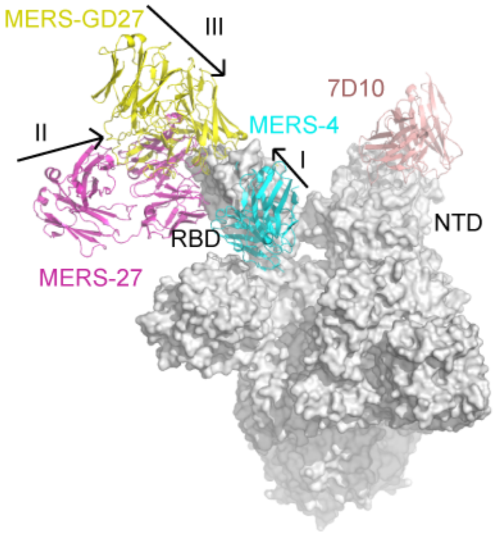

Supplementary Fig. 5 7D10 epitope outside the three categories of anti-RBD mAbs epitopes.

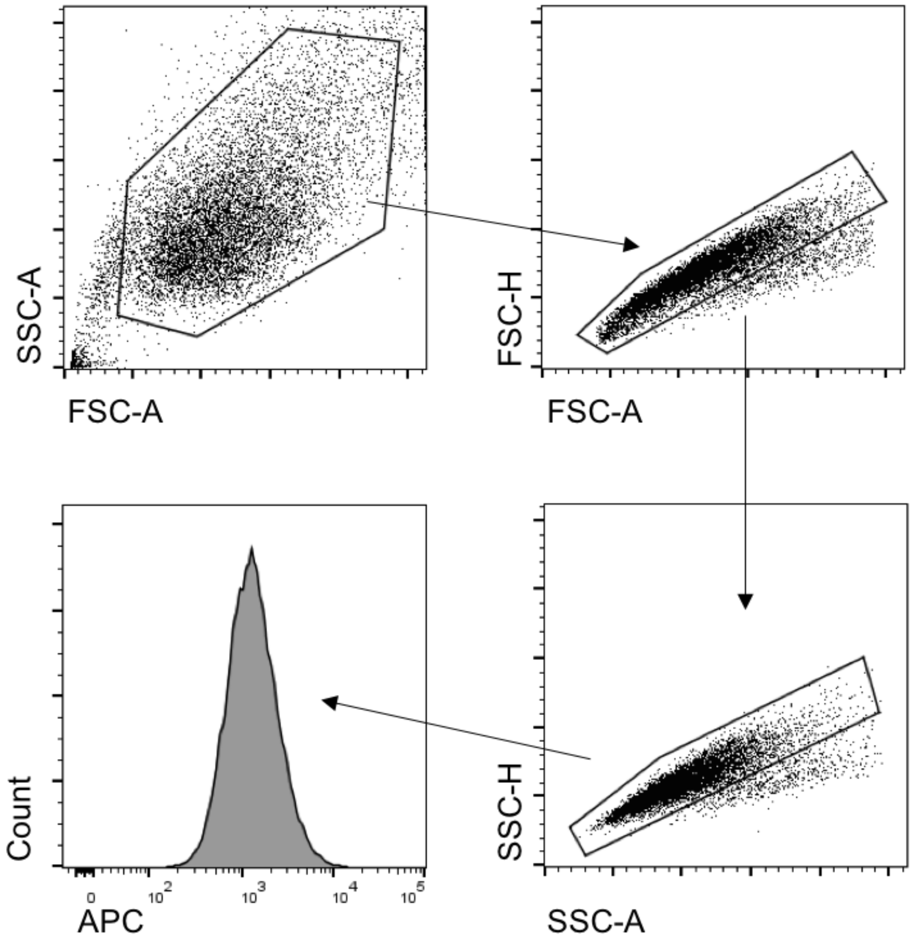

Supplementary Fig. 6 Gating strategy used for cell-surface staining analysis. All Huh7 cells which incubated with S or S and mAbs mixtures and stained with streptavidin APC used the same gating strategy (Figure 5 A).

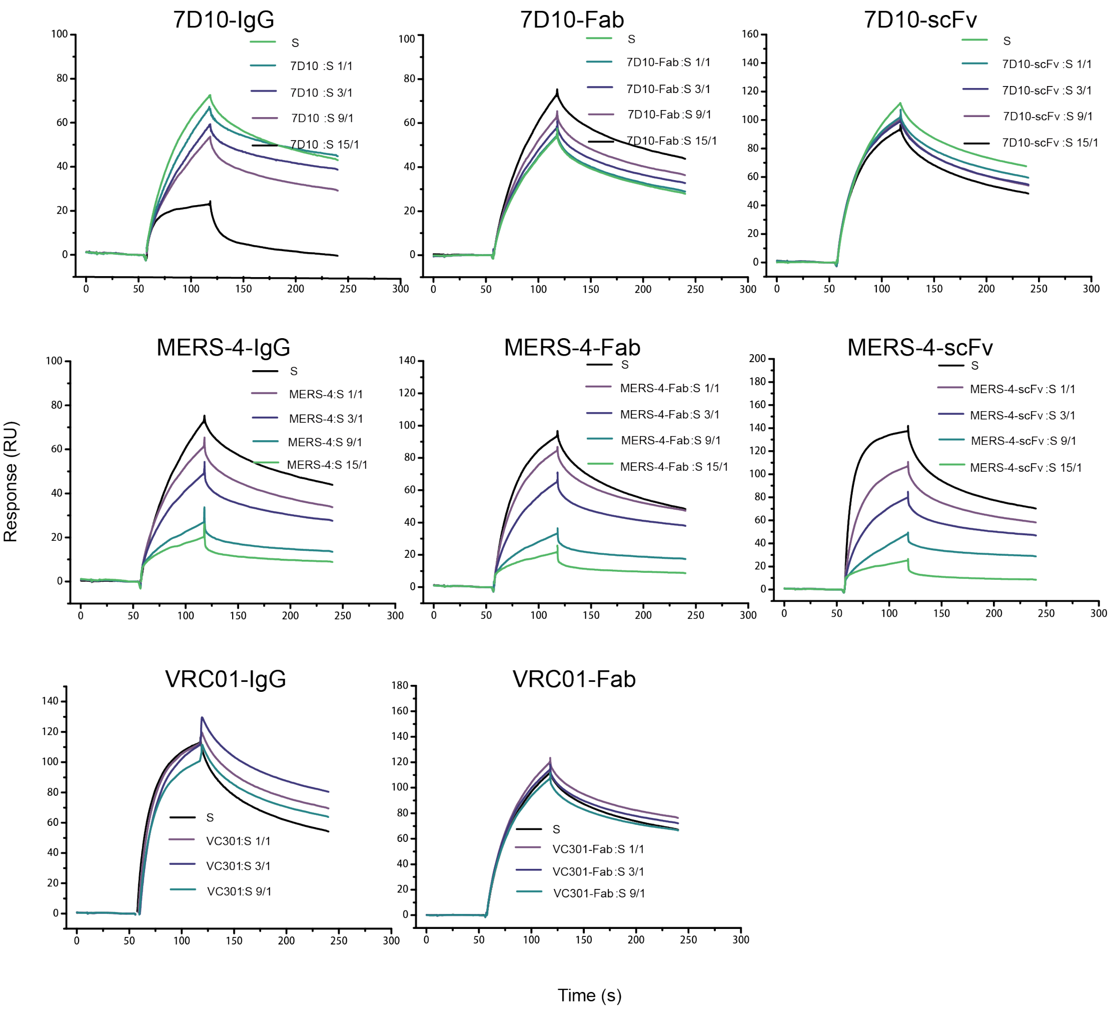

Supplementary Fig. 7 Binding of the soluble MERS spike trimer (S) to chip-immobilized DPP4 in the presence of 7D10-H measured by SPR. MERS-4 and VRC01 mAbs are used as positive control and unrelated control, respectively. Source data are provided as a Source Data file.

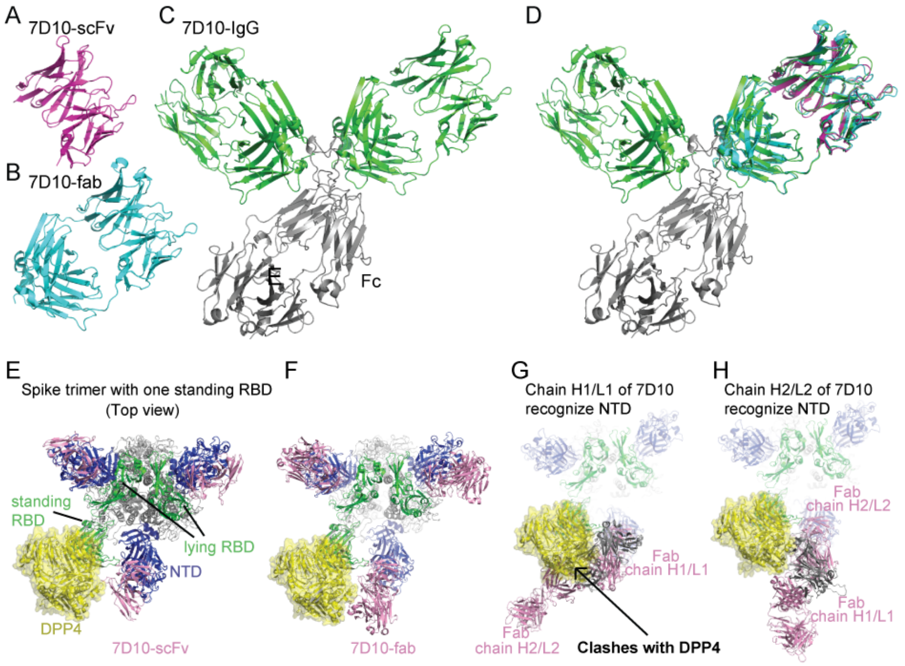

Supplementary Fig. 8 Models of 7D10-H IgG, Fab and scFv binding to the MERS-CoV spike trimer. (A) Crystal structure of 7D10-scFv was colored in pink. 7D10-Fab (B) and 7D10-IgG (C) were modeled by SWISS-MODEL with homologous modeling method. As for 7D10-IgG, Fab and Fc were colored in green and gray, respectively. (D) Structural superimposition of 7D10-scFv, 7D10-Fab and 7D10-IgG. Top view of structural superimpositions of the RBD/DPP4, NTD/7D10-scFv crystal structures (E) or modeled 7D10-Fab (F) or 7D10-IgG (G, H) onto the MERS-CoV S trimer glycoprotein in receptor-binding activated states with one RBD in the up positions (PDB: 5X5F [http://dx.doi.org/10.2210/pdb5X5F/pdb]). The spike trimer (RBD in green, NTD in blue and S2 subunit in gray) was shown as a cartoon. The DPP4 was shown with semi-transparent surface and colored in yellow. 7D10-scFv, 7D10-Fab and 7D10-IgG superimposition on the spike trimer were all colored pink.

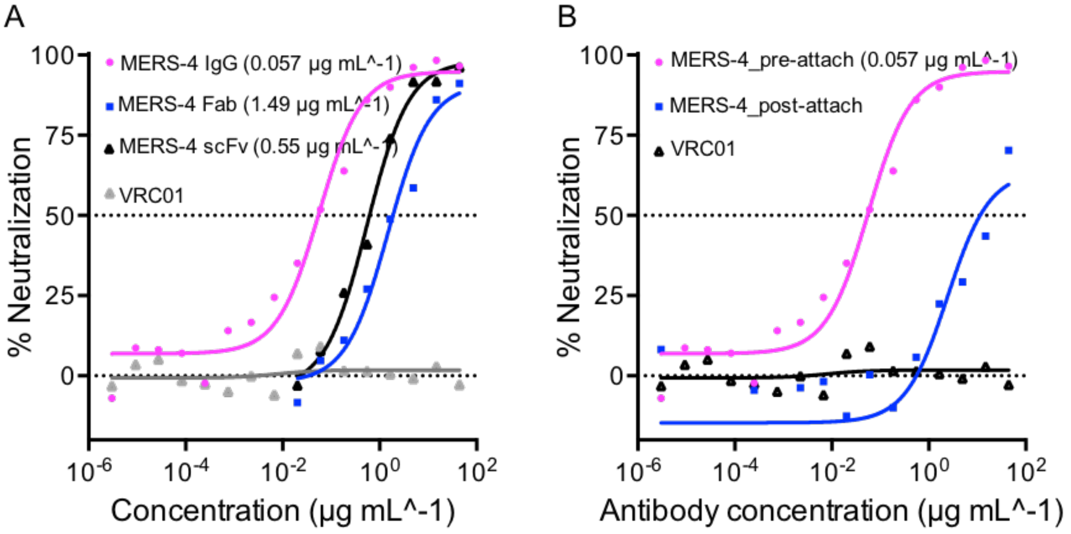

Supplementary Fig. 9 Neutralizing activities of RBD-specific mAb MERS-4. (A) Neutralizing test of MERS-4 scFv/Fab/IgG by pseudotyped MERS-CoV. (B) Post-attachment neutralizing activity of MERS-4. VRC01 was used as unrelated control. Source data are provided as a Source Data file.

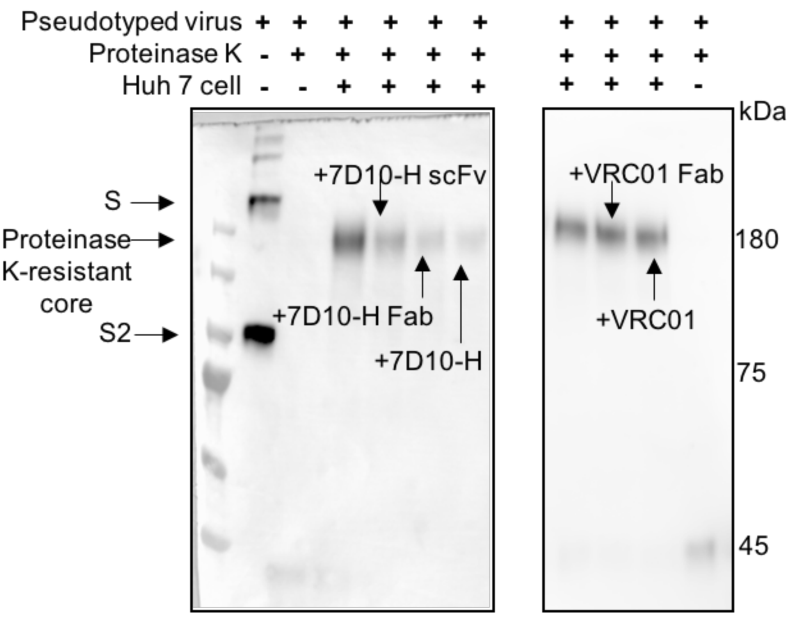

Supplementary Fig. 10 7D10-H inhibiting the transition to the post-fusion state of MERS-CoV S visualized by Western Blots. The effect of different forms of 7D10-H on the conformational change of the membrane-embedded S trimer on the MERS-CoV pseudovirus was probed by Western Blots using an anti-MERS-CoV S2 polyclonal antibody. Refolding to the post-fusion conformation was detected by the appearance of a proteinase-K resistant band. Proteinase K was used at 20 μg mL^-1. VRC01 was used as unrelated control. Digestion experiments and western blots were performed in triplicates, and a representative result is shown for each of them. Source data are provided as a Source Data file.

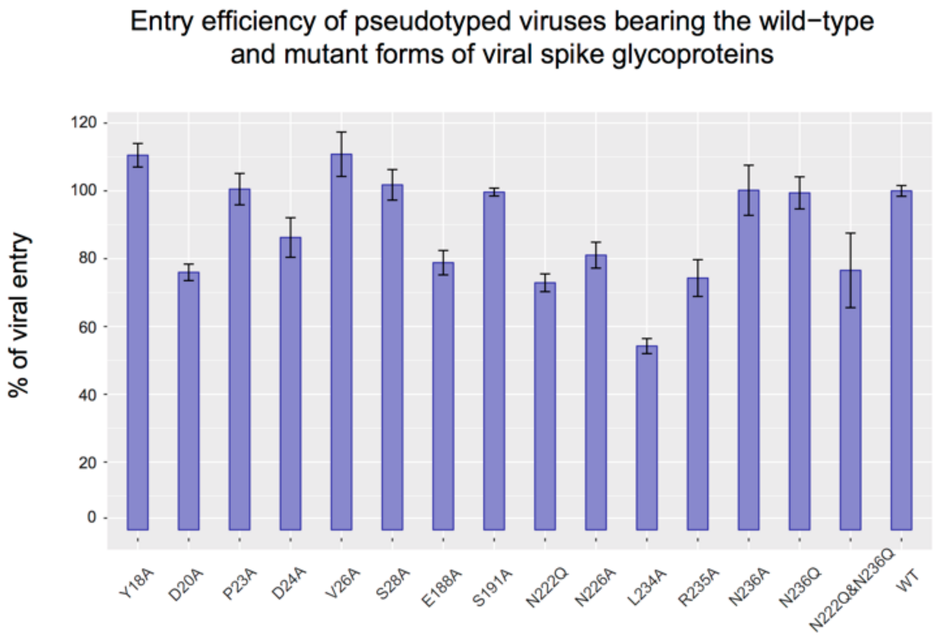

Supplementary Fig. 11 Infection efficiency of MERS-CoV pseudoviruses bearing wild type or mutant S into Huh7 cells. Data are shown as mean ± SD. Source data are provided as a Source Data file.

SUPPLEMENTARY TABLES

Supplementary Table 1. Sequence characters of 7D10

mAb		V segment	J segment	CDR1 sequence	CDR2 sequence	CDR3 sequence
7D10	H chain	IGHV1-12*01	IGHJ2*03	GYPFTSYN	IYPGNGDT	ARYGNYPSYAMDY
	L chain	IGKV3-12*01	IGKJ1*01,IGKJ1*02	KSVSASGYNY	LAF	QHSRDLPFT

Supplementary Table 2. Hydrogen bonds and salt bridges between the NTD and 7D10 scFv

	NTD	Distance[Å]	7D10
1	A: PRO23[N]	3.82	H: ASN101[OD1]
2	A: ASP24[O]	2.99	H: ASN33[ND2]
3	A: ASP24[OD2]	3.05	H: ARG[OH]
4	A: ASN226[OD1]	3.82	H: TYR52[OH]
5	A: ASN226[ND2]	3.63	H: TYR52[OH]
6	A: GLU188[OE2]	3.89	L: Arg121[NH1]
7	A: NAG508[O7]	2.55	H: TYR52[OH]
8	A: MAN519[O6]	2.79	H: ARG98[NH1]
9	A: MAN519[O6]	2.50	H: ARG98[NH2]

Supplementary Table 3. Contacts between Asn222-linked glycans and 7D10.

	NTD glycans	Distance[Å]	7D10
1	A: NAG508[O3]	3.05	H: SER31[OG]
2	A: NAG508[O3]	3.42	H: SER31[CB]
3	A: NAG508[N2]	3.63	H: SER31[OG]
4	A: NAG508[O7]	2.55	H: TYR52[OH]
5	A: NAG508[O7]	3.65	H: TYR52[CZ]
6	A: NAG508[C3]	3.99	H: SER31[OG]
7	A: NAG508[C7]	3.84	H: SER31[OG]
8	A: NAG508[C7]	3.15	H: TYR52[OH]
9	A: NAG508[C8]	3.87	H: SER31[OG]
10	A: NAG508[C8]	3.07	H: TYR52[OH]
11	A: NAG509[O6]	3.95	H: SER31[OG]
12	A: NAG509[O6]	3.78	H: TYR32[OH]
13	A: NAG509[O6]	3.70	H: TYR32[CE1]
14	A: NAG509[C6]	3.96	H: SER31[OG]
15	A: MAN519[O6]	3.67	H: TYR32[OH]
16	A: MAN519[O6]	2.79	H: ARG98[NH1]
17	A: MAN519[O6]	2.50	H: ARG98[NH2]
18	A: MAN519[O6]	3.02	H: ARG98[CZ]
19	A: MAN519[C6]	3.91	H: ARG98[NH1]
20	A: MAN519[C6]	3.78	H: ARG98[NH2]

Supplementary Table 4. Fold changes in MFI (median fluorescence intensity) of S with scFv/Fab/IgG relative to S alone.

I	MFI	Fold Change	II	MFI	Fold Change	III	MFI	Fold Change
Huh7 cells (NC)	93	-	Huh7 cells (NC)	92	-	Huh7 cells (NC)	109	-
S（PC）	2728	1	S（PC）	2632	1	S（PC）	1499	1
7D10-IgG:S 1/1	2596	0.95	7D10-Fab:S 1/1	2654	1.01	MERS-4-IgG:S 1/1	1238	0.83
7D10-IgG:S 3/1	1877	0.69	7D10-Fab:S 3/1	2556	0.97	MERS-4-IgG:S 3/1	741	0.49
7D10-IgG:S 9/1	1355	0.50	7D10-Fab:S 9/1	2493	0.95	MERS-4-IgG:S 9/1	220	0.15
7D10-IgG:S 27/1	1149	0.42	7D10-Fab:S 27/1	2101	0.80	MERS-4-IgG:S 27/1	151	0.10
7D10-scFv:S 1/1	2590	0.95	MERS-4-scFv:S 1/1	2262	0.86	MERS-4-Fab:S 1/1	1364	0.91
7D10-scFv:S 3/1	2608	0.96	MERS-4-scFv:S 3/1	2227	0.85	MERS-4-Fab:S 3/1	874	0.58
7D10-scFv:S 9/1	2428	0.89	MERS-4-scFv:S 9/1	962	0.37	MERS-4-Fab:S 9/1	747	0.50
7D10-scFv:S 27/1	2483	0.91	MERS-4-scFv:S 27/1	336	0.13	MERS-4-Fab:S 27/1	177	0.12
VRC01-IgG:S 1/1	2667	0.98	VRC01-Fab:S 1/1	2843	1.08			
VRC01-IgG:S 3/1	2657	0.97	VRC01-Fab:S 3/1	2821	1.07			
VRC01-IgG:S 9/1	2422	0.89	VRC01-Fab:S 9/1	2804	1.07			
VRC01-IgG:S 27/1	2427	0.89	VRC01-Fab:S 27/1	2615	0.99			

Supplementary Table 5. Summary of MERS-CoV S NTD-targeting neutralizing mAbs

mAbs	Species	Pseudotyped MERS-CoV (EMC) neutralization	Live MERS-CoV (EMC) neutralization	*In vivo* protection	Crystal structures available
7D10	Mouse	The IC_{50} was 0.18 µg/ml and the IC_{50} of 7D10-H was 0.06 µg mL^-1.	The PRNT IC_{50} was 0.2 µg mL^-1.	7D10-H can inhibit the infection of pseudotyped MERS-CoV in R26-hDPP4 mice.	Yes, NTD/7D10 scFv
5F9	Mouse	The IC_{50} was 0.24 µg mL^-1.	The PRNT IC_{50} was 0.2 µg mL^-1.	Not reported	Not reported
G2	Mouse	The IC_{50} was 0.010 µg mL^-1.	Not reported	G2 can protect against MERS-CoV infection in DPP4-transgenic mice.	Not reported
JC57-13	Rhesus macaques	The IC_{50} was 0.0085 µg mL^-1 (also showed as 0.068 µg mL^-1).	The PRNT IC_{50} was not available (N/A), but 50% neutralization was obtained at the concentration of 0.0032 µg mL^-1.	Not reported	Not reported
FIB-H1	Rhesus macaques	The IC_{50} was 0.0083 µg mL^-1.	Not reported	Not reported	Not reported
1.10f3	Transgenic H2L2 mouse	The IC_{50} was > 10 µg mL^-1.	The PRNT IC_{50} was > 1 µg mL^-1.	1.10f3 can partially protect from mortality (40%) in K18-hDPP4 mice.	Not reported

PRNT: plaque reduction neutralization testing

Transgenic H2L2 mouse: encoding the human immunoglobulin variable regions
