# Supplementary material for: Structural definition of a neutralization epitope on the N-terminal domain of MERS-CoV spike glycoprotein
Source: Nat Commun. 2019 Jul 11;10:3068. doi: 10.1038/s41467-019-10897-4 (PMC6624210; doi:10.1038/s41467-019-10897-4)
Supplement: Supplementary file 3 — Reporting Summary [file 41467_2019_10897_MOESM3_ESM.pdf]

nature research

Corresponding author(s): Wenjie Tan and Xinquan Wang

Last updated by author(s): May 22, 2019

Reporting Summary

Nature Research wishes to improve the reproducibility of the work that we publish. This form provides structure for consistency and transparency in reporting. For further information on Nature Research policies, see Authors & Referees and the Editorial Policy Checklist.

Statistics

For all statistical analyses, confirm that the following items are present in the figure legend, table legend, main text, or Methods section.

n/a	Confirmed	
☐	☒	The exact sample size (*n*) for each experimental group/condition, given as a discrete number and unit of measurement
☐	☒	A statement on whether measurements were taken from distinct samples or whether the same sample was measured repeatedly
☒	☐	The statistical test(s) used AND whether they are one- or two-sided<br>*Only common tests should be described solely by name; describe more complex techniques in the Methods section.*
☒	☐	A description of all covariates tested
☒	☐	A description of any assumptions or corrections, such as tests of normality and adjustment for multiple comparisons
☒	☐	A full description of the statistical parameters including central tendency (e.g. means) or other basic estimates (e.g. regression coefficient) AND variation (e.g. standard deviation) or associated estimates of uncertainty (e.g. confidence intervals)
☒	☐	For null hypothesis testing, the test statistic (e.g. *F*, *t*, *r*) with confidence intervals, effect sizes, degrees of freedom and *P* value noted<br>*Give P values as exact values whenever suitable.*
☒	☐	For Bayesian analysis, information on the choice of priors and Markov chain Monte Carlo settings
☒	☐	For hierarchical and complex designs, identification of the appropriate level for tests and full reporting of outcomes
☒	☐	Estimates of effect sizes (e.g. Cohen's *d*, Pearson's *r*), indicating how they were calculated

Our web collection on statistics for biologists contains articles on many of the points above.

Software and code

Policy information about availability of computer code

Data collection	NCBI was used for downloading the published MERS-Spike sequences to do the mutation site analysis.
Data analysis	HKL2000, CCP4, COOT and PHNIX were used at the determination of complex structure for data processing, model building and refinement. PyMOL was used to generate the structural figures. GraphPad was used to do data analysis and make some of the figures.

For manuscripts utilizing custom algorithms or software that are central to the research but not yet described in published literature, software must be made available to editors/reviewers. We strongly encourage code deposition in a community repository (e.g. GitHub). See the Nature Research guidelines for submitting code & software for further information.

Data

Policy information about availability of data

All manuscripts must include a data availability statement. This statement should provide the following information, where applicable:

- Accession codes, unique identifiers, or web links for publicly available datasets
- A list of figures that have associated raw data
- A description of any restrictions on data availability

The source data underlying Figs 1A-D, 3, 4 and 6A-C and Supplementary Figs 1A-C, 2, 3, 5, 7, 8 and 9 are provided as a Source Data file. Crystal structure presented in this work has been deposited in the Protein Data Bank (PDB) and are available with accession codes 6J11.

Field-specific reporting

Please select the one below that is the best fit for your research. If you are not sure, read the appropriate sections before making your selection.

☒ Life sciences ☐ Behavioural & social sciences ☐ Ecological, evolutionary & environmental sciences

For a reference copy of the document with all sections, see nature.com/documents/nr-reporting-summary-flat.pdf

Life sciences study design

All studies must disclose on these points even when the disclosure is negative.

Sample size	The total number of R26-hDPP4 mouse model was 17. Protection with 7D10-H or MERS-4 each had 5 mice. Control with 3C11 or PBS had 3 and 4 mice, respectively.
Data exclusions	No data excluded
Replication	All the binding and neutralizing assays were performed in duplicate. All attempts at replication were successful.
Randomization	All mice from the Institute for Laboratory Animal Resources, National Institute for Food and Drug Control (Beijing, China) were randomly grouped.
Blinding	N/A

Reporting for specific materials, systems and methods

We require information from authors about some types of materials, experimental systems and methods used in many studies. Here, indicate whether each material, system or method listed is relevant to your study. If you are not sure if a list item applies to your research, read the appropriate section before selecting a response.

Materials & experimental systems

n/a	Involved in the study	
☐	☒	Antibodies
☐	☒	Eukaryotic cell lines
☒	☐	Palaeontology
☐	☒	Animals and other organisms
☒	☐	Human research participants
☒	☐	Clinical data

Methods

n/a	Involved in the study	
☒	☐	ChIP-seq
☐	☒	Flow cytometry
☒	☐	MRI-based neuroimaging

Antibodies

Antibodies used	Rabbit anti MERS-CoV S2 polyclonal antibody, used for Western Blots, ThermoFisher, USA, Cat#PA5-81788.<br>HRP goat anti-rabbit secondary antibody, used for Western Blots, HuaxingBio, China, Cat#HX2027.<br>MERS-4, MERS-27, MERS-GD27, VRC01 and 3C11 were produced in our lab by 293T cell line.
Validation	MERS-4, MERS-27 were human monoclonal antibody sorted from the non-immune human ScFv yeast library, cited paper:Jiang L, Wang N, Zuo T, Shi X, Poon KM, Wu Y, et al. Potent neutralization of MERS-CoV by human neutralizing monoclonal antibodies to the viral spike glycoprotein. Sci Transl Med. 2014;6(234):234ra59. Epub 2014/04/30. doi: 10.1126/scitranslmed.3008140. PubMed PMID: 24778414.<br>MERS-GD27 was a human monoclonal antibody sorted from MERS-CoV survivor, cited paper: Niu P, Zhang S, Zhou P, Huang B, Deng Y, Qin K, et al. Ultrapotent Human Neutralizing Antibody Repertoires Against Middle East Respiratory Syndrome Coronavirus From a Recovered Patient. J Infect Dis. 2018;218(8):1249-60. Epub 2018/05/31. doi: 10.1093/infdis/jiy311. PubMed PMID: 29846635.<br>VRC01 was a HIV-1 mAb, as the negative control in this study, cited paper: X. Wu, Z.Y. Yang, Y. Li, C.M. Hogerkorp, W.R. Schief, M.S. Seaman, T. Zhou, S.D. Schmidt, L. Wu, L. Xu, et al. Rational design of envelope identifies broadly neutralizing human monoclonal antibodies to HIV-1 Science, 329 (2010), pp. 856-861. PubMed PMID: 20616233.<br>3C11 was a H5N1 mAb, as the negative control in this study, cited paper: Zhou, B., Zhong, N., and Guan, Y. (2007) Treatment with convalescent plasma for influenza A (H5N1) infection. N. Engl. J. Med. 357, 1450 –1451. PubMed PMID: 17914053

Eukaryotic cell lines

Policy information about cell lines

Cell line source(s)	HEK293, 293T, Huh7 and Vero E6 cells were bought from ATCC.

Authentication	293T cells, referred to website:https://www.atcc.org/Products/All/CRL-3216.aspx<br>HEK293, referred to website: https://www.atcc.org/products/all/CRL-1573.aspx<br>Vero E6, referred to website: https://www.atcc.org/products/all/CRL-1586.aspx<br>Huh7, referred to website:
Mycoplasma contamination	All cell lines were tested negative for mycoplasma contamination.
Commonly misidentified lines (See ICLAC register)	Not applicable

Animals and other organisms

Policy information about studies involving animals; ARRIVE guidelines recommended for reporting animal research

Laboratory animals	Female BALB/c mice aged six to eight weeks were used for mAb production. Genetically-modified R26-hDPP4 mice aged four weeks were used for protection assay.
Wild animals	This study didn't involve any wild anmals.
Field-collected samples	This study didn't collect any samples from the field.
Ethics oversight	All studies were performed in compliance with animal protocols (#2017-B-004) approved by the Institutional Animal Care and Use Committee of the National Institute for Food and Drug Control, China Food and Drug Administration (CFDA, Beijing, China) and in compliance with the “Guide for the Care and Use of Laboratory Animals” (National Academies Press: Washington, DC, USA, 2011; 8th ed.). The license number of the Animal Use Certificate issued by the Science & Technology Department of China (Beijing, China) was SYXK 2016-004, approved on 18 February 2016.

Note that full information on the approval of the study protocol must also be provided in the manuscript.

Flow Cytometry

Plots

Confirm that:

- [x] The axis labels state the marker and fluorochrome used (e.g. CD4-FITC).
- [x] The axis scales are clearly visible. Include numbers along axes only for bottom left plot of group (a 'group' is an analysis of identical markers).
- [ ] All plots are contour plots with outliers or pseudocolor plots.
- [ ] A numerical value for number of cells or percentage (with statistics) is provided.

Methodology

Sample preparation	The samples were Huh7 cells alone or incubation with the mix of soluble MERS-CoV S trimer and antibodies (7D10-H , MERS-4 and their Fabs, scFvs), then stained the cell surface with the Streptavidin-APC to run by flow cytometry.
Instrument	FACS Aria III (BD eBiosciences)
Software	FlowJo V10 was used to analyze the data.
Cell population abundance	In this assay, in total 20000 cells of each sample were collected.
Gating strategy	We used SSC-A/FSC-A, FSC-H/FSC-A and SSC-H/SSC-A to select single cells for each sample. No other gating strategy used in the cell surface staining assay.

- [x] Tick this box to confirm that a figure exemplifying the gating strategy is provided in the Supplementary Information.
